# Dairy Foods and Body Mass Index over 10-Year: Evidence from the Caerphilly Prospective Cohort Study

**DOI:** 10.3390/nu10101515

**Published:** 2018-10-16

**Authors:** Jing Guo, Anestis Dougkas, Peter C. Elwood, David I. Givens

**Affiliations:** 1 Institute for Food, Nutrition and Health, University of Reading, Reading RG6 6AR, UK; sarah.guo@reading.ac.uk; 2 Institut Paul Bocuse, Chateau Du Vivier, BP 25-69131 Ecully CEDEX, France; anestis.dougkas@institutpaulbocuse.com; 3 Department of Primary Care and Public Health, Cardiff University, Cardiff CF10 3AT, UK; peter.c.elwood@gmail.com

**Keywords:** dairy, milk, cheese, cream, butter, body mass index

## Abstract

The association between dairy product consumption and body mass index (BMI) remains controversial. The aim of the present study was to investigate the association between total dairy, milk, cheese, cream and butter consumption and BMI change over a 10-year follow-up by using long-term follow-up cohort data from the Caerphilly Prospective Cohort Study (CAPS). The CAPS included 2512 men aged 45–59 years at baseline, who were followed up at 5-year intervals for over 20-year. A semi-quantitative food frequency questionnaire estimated the intake of dairy consumption, including milk, cheese, cream and butter at baseline, 5-year and 10-year follow-up. In total, men free of cardiovascular disease, diabetes and cancer (*n* = 1690) were included in current analysis. General linear regression and logistic regression were used for data analysis. The results showed higher cheese consumption was associated with lower BMI at the 5-year follow-up (*p* = 0.013). There was no evidence that higher consumption of total dairy, milk, cream and butter were significantly associated with BMI during the over the 10-year following-up. This study suggest that cheese consumption have beneficial effects on lowering BMI, which needs further investigation.

## 1. Introduction

The prevalence of obesity has reached epidemic proportions with more than 600 million adults worldwide classified as clinically obese (body mass index (BMI) ≥ 30 kg/m^2^) [1]. Among the various approaches to tackle obesity and its comorbidities, a heathy diet is one of the key determinants of reducing obesity rates [2]. Given that dairy products are naturally rich in protein and essential micronutrients, including calcium, potassium and vitamin A, they are recommended as an integral part of a healthy diet by many countries [3]. However, dairy is also a major contributor to saturated fatty acids (SFA) and energy intake [4], thus their role in development of obesity has been questioned and explored by several studies [5]. 

Evidence from a meta-analysis of 29 randomized controlled trials (RCTs), including a total of 2101 women and men, showed that higher dairy consumption was inversely associated with weight change in short-term (<1 year) and energy-restricted trials, but not in long term studies (≥1 year and ≤2 year) or ad libitum studies [6]. Although several cross-sectional studies have shown dairy consumption to be inversely associated with body weight [7], findings on the association between dairy consumption and body weight in long-term studies (>2 year) are inconsistent and limited [8,9,10]. Milk consumption was found to be inversely associated with weight change in long-term studies of Vergnaud et al. [9] and Rosell et al. [8], but not in that of Mozaffarian et al. [10]. Furthermore, the study of Rosell et al. [8] indicated that the association between dairy consumption and weight change depends on the different types of dairy product. As BMI is widely used as an indicator of overweight and obesity [11], we therefore aimed to prospectively investigate the association between consumption of different dairy products and changes of BMI over 10-year follow-up in the Caerphilly Prospective Study.

## 2. Material and Methods

### 2.1. Study Population

The Caerphilly prospective cohort study (CAPs) initially recruited 2512 men aged 45–59 years at baseline (Phase 1: 1979–1983), which represented 89% of the age group living in Caerphilly (Wales, UK) and surrounding villages and were seen at 5-year intervals and followed up for over 20-years. At Phase 2 (1984–1988), 561 men being lost to follow-up and an additional 447 men in the same age range (50–64 years) were recruited to provide a total 2398 men. At baseline, the men were invited to complete a general health questionnaire and a food frequency questionnaire, and anthropometric measurements were taken every 5-year intervals. Details of the methods have been reported in detail elsewhere [12,13,14,15]. Ethical approval was received from the South Glamorgan Ethics Committee and all individuals provided written informed consent prior to commencing the study. 

### 2.2. Dairy Product Consumption

The men were asked to complete a semi-quantitative food frequency questionnaire (FFQ) with their partners, which included estimation of the mean daily consumptions of 50 food items typical for the British diet at baseline, 5-year and 10-year. Dairy consumption included questions about milk, cheese, cream and butter intake [16,17]. In terms of milk intake, participants chose from four possible daily intake options: None; less than ½ pint; between ½ pint and 1 pint, and more than 1 pint. We converted the pints to gram by using standard units of 585 g for 1 pint of milk [18]. In terms of the cheese, butter, and cream consumed, participants had to record the amount of each dairy product that was consumed by the family per week. Based on the number of persons (both adults and children) per household, a family size variable was created in order to calculate dairy intake per person per day. In this derived variable, adults and older children aged 5 to 16 years were counted as one, younger children aged 1 to 4 years were counted as half and infants were excluded [19]. The validity of the FFQ was tested using data from a 7-day weighed dietary intake record that was kept by 30% of the men in Phase 1, results have been described previously in detail [20,21]. Briefly, there was a statistically significant correction between methods for all food items ranging from 0.3 to 0.5 (milk: 0.42, *p* < 0.001; cheese: 0.32, *p* < 0.001; cream: 0.30, *p* < 0.001; butter: 0.45, *p* < 0.001) [20,21]. For consumption of dairy products (g/day), the weight of milk, cheese, butter and cream were summed.

### 2.3. BMI and Other Covariates

Height and weight were measured at were available at each examination during the 10-year follow-up [17]. BMI data (kg/m^2^) was calculated by weight divided by height squared [22]. Several lifestyle and dietary factors, which could confound the analysis of dairy and BMI relationships, were either available or calculated [16]. These included age, social class, smoking habits, leisure activity and other dietary variables, such as total energy intake and intakes of alcohol, eggs, red meat and sugar. Social class was based upon the men’s most recent occupation [23], and was categorised into manual and non-manual; Smoking habits were defined at three levels: Never smoked, former smokers, and current smokers [16]; Leisure activity was categorised into yes and no for any heavy work or exercise was done in the leisure time in the last 7 days before subjects taking FFQ [16]. 

### 2.4. Statistical Analyses

In order to ensure consistency of the subject group throughout the study, the 561 men who dropped out after Phase 1 were excluded from this analysis. In addition, subjects who previously had cardiovascular disease and/or diabetes and/or cancer (*n* = 101) and subjects with missing confounding factor data (*n* = 160) were excluded. Therefore, a total 1690 men were available for the current analysis. 

Data were analysed using Stata version 15.0 (STATA Corporation, College Station, TX, USA). As the semi-quantitative food frequency questionnaire has been collected at first 15 years, thus, the associations of dairy intake (total dairy, milk, cheese, cream and butter) of the baseline with baseline BMI were investigated as cross-sectional analysis, and the associations of the dairy intake (total dairy, milk, cheese, cream and butter) of the baseline with BMI changes at 5-year and 10-year as longitudinal analyses. Subjects were categorised into quartiles for total dairy products, cheese and butter consumption and into groups as specified on FFQ for milk. Because a high proportion of subjects that did not consume cream (*n* = 1105), subjects were categorised in tertiles for cream consumption. 

Logistic regression and general linear regression were used to investigate the associations of dairy products and categorical and continuous variables, respectively. Multivariable models for continuous and discrete data were adjusted for a number of dietary and lifestyle variables. These confounding variables were selected if significantly associated in simple correlations, or if known to be correlated from previously published studies [24]. For the cross-sectional analysis, the first multivariate-adjusted model for all analyses included the confounding factors of age (years), social class (manual and non-manual workers), alcohol intake (as ethanol, mL/week), smokers (non-smoker, current smoker, previous smoker), leisure activity (yes and no), total energy intake (MJ/day). The second multivariate-adjusted model was further adjusted for sugar intake (g/day), eggs intake (number/week) and red meat consumption (times/week). For the longitudinal analysis, baseline BMI was added into the first and second multivariate-adjusted models as multivariate model 3 and model 4, respectively. Furthermore, the diagnosis of chronic diseases may lead to weight changes and then confound the associations between dairy consumption and BMI change. Therefore, we have further adjusted for diagnosed diabetes, cancer, cardiovascular disease, and hypertension in the model 4. In addition, we conducted additional multivariate analyses stratified by baseline BMI and total energy intake. Pattern of consumption of dairy products over 10-years was analysed. Paired *t*-test was used to determine the significance of dairy consumption between baseline and 5-year or 10-year. Results were considered statistically significant at *p* = 0.05 or less.

## 3. Results

The baseline characteristics of 1690 subjects were described in Table 1. Subjects in the highest quartiles of total dairy intake were significantly more likely to be smokers (*p* = 0.024) and had a higher total energy intake (*p* < 0.001), sugar intake (*p* < 0.001), eggs intake (*p* < 0.001) or red meat consumption (*p* = 0.001). There were no associations between total dairy intake and age, social class, leisure activity, alcohol intake, fish intake, fruit or vegetable consumption. After controlling for total energy intake, subjects with the highest dairy consumption tended to have a higher intake of fat (*p* < 0.001), but not protein or fibre (cereal or vegetable sources).

The association of total dairy, milk, cheese, cream and butter intakes with BMI at baseline and BMI changes from baseline are shown in Table 2, Table 3, Table 4, Table 5 and Table 6, respectively. Higher total dairy consumption was not significantly associated with BMI cross-sectionally or with BMI changes during the 10-year follow-up (Table 2).

Milk intake was inversely associated with BMI cross-sectionally (*p* = 0.007, Table 3), but there were no associations between milk intake and BMI changes during the 10-year follow-up. Cheese consumption was significantly associated with higher BMI at baseline (*p* = 0.045, Table 4), however, higher cheese intake at baseline was associated with lower BMI change (*p* = 0.013) at the 5-year follow-up, but not at the 10-year. Cream intake (Table 5) and butter intake (Table 6) were not associated with BMI at both cross-sectional and longitudinal examinations. Furthermore, stratified analyses showed that there was no evidence for a statistical interaction (*p*-interaction > 0.05) of baseline total energy intake and BMI for the associations between dairy consumption (total dairy, milk, cheese, cream and butter) and BMI at baseline or BMI changes from the baseline during the follow-up. Consumption of total dairy, milk, cream and butter continuously decreased over the 10-years following-up. Cheese was relative stable at the first 5-year, but significantly decreased at the 10-year examination (Online Supplemental Table S1).

## 4. Discussion

Our main findings demonstrate that higher consumption of total dairy, milk, cheese cream and butter is not associated with BMI change over the 10-year. Interestingly, we found that higher cheese intake was inversely associated with BMI change at 5-year examination. 

No associations were found between total dairy and milk consumption with BMI changes over 10-years in the current study, which is in agreement with results from previous studies [9,10,25]. Specifically, Mozaffarian et al. [10] reported neutral associations of milk consumption with BMI changes by including three cohort studies (*n* = 120,877, men and women) with follow-up periods from 12 years to 20 years. Studies of Samara et al. [25] and Vergnaud et al. [9] also reported no association between total dairy with 5-year and 6-year BMI changes, respectively. In contrast, Rosell et al. [8] found whole milk and sour milk consumption were inversely associated with weight change over a 9-year follow-up. However, Rosell et al. [8] did not analyse the data for whole milk and sour milk separately. 

In the current study, we observed an inverse association between cheese consumption and BMI change after 5-year. Our result is consistent with results of Rosell et al. [8], which also found an inverse association between cheese consumption and BMI change in 19,352 Swedish women aged 40–55 years at baseline over 9-year follow-up. However, Samara et al. [25] and Vergnaud et al. [9] reported neutral associations between cheese consumption and body weight change after 5-year and 6-year follow-up periods, respectively. Previous studies [9,26] indicated the relationship of dairy consumption and body weight change may differ, due to gender, age, and baseline weight of the subjects. Therefore, the difference in the observed associations of cheese intake and BMI changes between the current study and Samara et al. [25] or Vergnaud et al. [9] may be due to characteristics differences of the investigated subjects. In addition, studis of Samara et al. [25] and Vergnaud et al. [9] included both men and women for the analysis, whereas only men were included in the current study. In addition, subjects in the study of Samara et al. [25] had a wider age range of 28–60 years compared to current study of 45–59 years at baseline. In the current study, the inverse association of cheese intake and BMI change disappeared at 10-year, which may be because cheese intake was significantly decreased after 5-year. 

Low-fat dairy has been recommended as healthy choices recently [27], however, there is little evidence to show a detrimental effect of high-fat dairy on health outcomes. In the current study, we found no association between cream and butter consumption and BMI change during the 10-year following up. Our study was in line with recently meta-analyses [28,29], which also reported a neutral association between high-fat dairy or butter consumption with mortality, cardiovascular disease (CVD) and diabetes. Therefore, future studies are needed to investigate further on the effect of the so-called dairy matrix on health effects. 

Numerous plausible mechanisms underlying the potential beneficial effect of dairy consumption on body weight have been suggested. As regards dairy components, calcium has received the greatest attention, with calcium intake shown to be inversely associated with body weight in a number of observational studies [30,31,32,33]. A recently meta-analysis [34] of both RCTs and observational studies showed increasing calcium consumption can reduce body weight in subjects who have a normal BMI. Proposed mechanisms suggest that calcium increases fat oxidation, thermogenesis, faecal fat excretion, energy expenditure, bile acid excretion and decreases fat absorption and lipogenesis [30,35,36,37]. In addition, dairy constituents, such as lactose, proteins, and medium chain fatty acids, have been proposed to impact on body weight through regulation of food intake or macronutrient metabolism [38,39,40,41]. It is not known why the inverse association was only found in the cheese not the other types of dairy in the current study, perhaps it is associated with fermented process of the cheese production [3]. Evidence from RCTs [42] suggests a beneficial effect of fermented dairy consumption on gut homeostasis. Furthermore, findings from a recent meta-analysis [28] of 29 prospective cohort studies showed that cheese intake was associated with lower risk of CVD risk and mortality. Thus, the mechanism of higher cheese consumption on reduction of body weight and CVD risk needs to be elucidated in further studies. Overall, the inverse association between cheese consumption and BMI needs to be further explored in large cohort studies and long-term RCTs.

The major strength of the CAPs study is the long-term follow up period for different types of dairy consumption over 10-years, which could provide sufficient evidence on the association between different types of dairy consumption and BMI changes. However, a limitation of the current study is the observational nature of the approach, which does not allow determination of a cause and effect association. Residual confounding factors may have influenced the association between diet and BMI [43]. In addition, only men were recruited for the CAPs study, thus, which cannot represent for the whole population. Furthermore, yogurt consumption was reported to associate with lower body mass index [44], but yogurt consumption was not collected in the current FFQ. It is well known that self-reported food frequency questionnaires are prone to a number of limitations and errors, and particularly due to the fact that not every possible food and dairy product is included in the questionnaire [45]. For example, in the present study the fat contents of the dairy products was not specified. However, based on the 7-day weighed dietary intake, semi-skimmed milk consumption was reported by only 11% of subjects (*n* = 70), thus we assume that the predominant milk consumed at the time of the analysis was whole milk. Finally, evidence in our study agreed with previous study that the pattern of consumption of dairy products may vary over time [46], which is another limitation to investigate the long-term effect (e.g., >10 years) of dairy consumption on human health (e.g., weight management, CVD and diabetes risk).

## 5. Conclusions

In conclusion, our study showed higher cheese consumption was associated with lower BMI. Further evidence from human intervention studies is needed to better understand the role of cheese in bodyweight control and the mechanisms involved.

## Figures and Tables

**Table 1:** Background characteristics according to quartile of baseline total dairy product intake.

Characteristics	Total Dairy Intake (g/day) *								*p* for Trend
	0 ≤ Dairy ≤ 183		183 < Dairy < 240		240 ≤ Dairy < 485		485 ≤ Dairy ≤ 842		
	Mean	SD	Mean	SD	Mean	SD	Mean	SD	
Participants, *n*	423		421		424		422		
Milk, g/day	115.0	60.7	146.3	10.1	413.5	82.5	516.8	129.5	
Cheese, g/day	15.2	12.1	20.4	11.7	15.9	12.5	22.4	13.5	
Cream, g/day	0.8	2.0	2.0	4.0	1.4	2.8	2.8	4.9	
Butter, g/day	11.0	13.2	32.2	14.8	18.8	20.9	35.9	18.5	
Age, year	52	4.4	52	4.3	51	4.5	52	4.5	0.516
Manual workers, %	62.4		67.7		61.3		64.7		0.964
Leisure activity, %	48.7		45.6		47.2		49.5		0.663
Current smokers, %	48.0		51.5		52.4		55.9		0.024
Alcohol intake (ethanol), mL/week	32.5	34.9	33.3	35.9	27.0	32.1	31.1	43.8	0.116
Total energy intake, MJ/day	8.6	2.3	9.7	2.1	9.4	2	10.7	2.5	<0.001
Fibre (cereal sources), g/day	7.6	4.7	7.6	4.2	8	4.6	8.1	4.7	0.084
Fibre (vegetable sources), g/day	8.4	2.8	8.4	2.6	8.6	2.7	8.5	2.8	0.259
Protein, % of food energy	13.1	2.2	12.7	1.8	13.6	1.9	13	1.9	0.198
Fat, % of food energy	37.8	7.0	40.3	6.9	38.9	6.7	42.1	6.8	<0.001
Sugar, g/day	81.9	32.8	91.2	34.1	93.0	31.2	110.7	41.2	<0.001
Eggs, *n*/week	3.5	2.5	3.8	2.2	3.8	2.6	4.7	5.4	<0.001
Fruit, times/week	5.3	4.9	5.3	4.5	4.9	4.2	4.9	4.4	0.396
Vegetable, times/week	9.4	4.6	9.2	4.3	9.6	4.4	9.5	4.4	0.471
Red meat, times/week	5.2	2.5	5.5	2.9	5.4	2.6	5.9	2.9	0.001
Fish, times/week	0.9	0.8	1.1	0.9	1.0	0.8	1	0.8	0.144

SD: Standard Deviation; * Total dairy includes milk, cheese, cream and butter intake.

**Table 2:** Cross-sectional analysis of body mass index (BMI) at baseline and longitudinal analyses over 10-year of BMI changes from baseline by quartile of total dairy intake.

	Total Dairy Intake (g/day) *								*p* for Trend
	0 ≤ Dairy ≤ 183		183 < Dairy < 240		240 ≤ Dairy < 485		485 < Dairy ≤ 842		
Cross-sectional analysis									
Participants, *n*	419		417		421		417		
BMI mean (SD), kg/m^2^	26.4	3.8	26.6	3.4	26.2	3.4	25.8	3.3	
Unadjusted Coef. (SE)	1 (reference)		0.186	0.240	−0.217	0.239	−0.601	0.240	0.004
Multivariate model 1 Coef. (SE) ^†^	1 (reference)		0.233	0.240	−0.116	0.240	−0.388	0.251	0.055
Multivariate model 2 Coef. (SE) ^‡^	1 (reference)		−0.315	0.253	−0.100	0.240	−0.315	0.253	0.120
Longitudinal analysis at 5-years									
Participants, *n*	415		414		418		415		
BMI mean change from baseline (SD), kg/m^2^	0.3	1.5	0.3	1.6	0.2	1.4	0.2	1.3	
Unadjusted Coef. (SE)	1 (reference)		0.002	0.102	−0.031	0.102	−0.092	0.102	0.342
Multivariate model 3 Coef. (SE) ^§^	1 (reference)		0.007	0.103	−0.054	0.103	−0.167	0.108	0.100
Multivariate model 4 Coef. (SE) ^‖^	1 (reference)		−0.003	0.104	−0.051	0.104	−0.156	0.110	0.136
Longitudinal analysis at 10-years									
Participants, *n*	337		353		354		360		
BMI mean change from baseline (SD), kg/m^2^	0.5	1.9	0.5	2.0	0.4	1.9	0.4	1.7	
Unadjusted Coef. (SE)	1 (reference)		0.041	0.143	−0.107	0.143	−0.017	0.143	0.661
Multivariate model 3 Coef. (SE) ^§^	1 (reference)		0.021	0.143	−0.175	0.143	−0.129	0.150	0.197
Multivariate model 4 Coef. (SE) ^‖^	1 (reference)		0.001	0.143	−0.186	0.144	−0.126	0.152	0.214

SD: Standard Deviation; SE: Standard Error; * Total dairy includes milk, cheese, cream and butter intake. ^†^ Model 1: Multivariable-adjusted model adjusted for age, social class (manual and non-manual workers), alcohol intake (non-drinker, drinker has been divided into 3 equal groups), smokers (non-smoker, current smoker, previous smoker), leisure activity (yes and no), total energy intake. ^‡^ Model 2: Model 1 and additionally adjusted for sugar intake, red meat intake and egg intake. ^§^ Model 3: Model 1 and additionally adjusted for BMI at baseline. ^‖^ Model 4: Model 2 and additionally adjusted for BMI at baseline, incident of diabetes, cancer, hypertension and cardiovascular disease during 10-year following up.

**Table 3:** Cross-sectional analysis of BMI at baseline and longitudinal analyses over 10-year of BMI changes from baseline by quartile of milk intake.

	Milk Intake (g/day)								*p* for Trend
	0		0 < Milk ≤ 293		293 < Milk ≤ 585		585 ≤ Milk		
Cross-sectional analysis									
Participants, *n*	93		779		692		110		
BMI mean (SD), kg/m^2^	27.3	4.4	26.4	3.5	26.1	3.4	25.4	3.3	
Unadjusted Coef. (SE)	1 (reference)		−0.907	0.380	−1.181	0.383	−1.846	0.488	<0.001
Multivariate model 1 Coef. (SE) *	1 (reference)		−0.926	0.375	−1.096	0.380	−1.708	0.486	0.001
Multivariate model 2 Coef. (SE) ^†^	1 (reference)		−0.896	0.377	−1.023	0.383	−1.540	0.492	0.007
Longitudinal analysis at 5-years									
Participants, *n*	92		773		687		110		
BMI mean change from baseline (SD), kg/m^2^	0.1	1.8	0.3	1.5	0.2	1.3	0.3	1.6	
Unadjusted Coef. (SE)	1 (reference)		0.212	0.163	0.124	0.164	0.231	0.208	0.983
Multivariate model 3 Coef. (SE) ^‡^	1 (reference)		0.190	0.162	0.069	0.164	0.130	0.210	0.557
Multivariate model 4 Coef. (SE) ^§^	1 (reference)		0.135	0.164	0.041	0.166	0.106	0.213	0.664
Longitudinal analysis at 10-years									
Participants, *n*	68		648		595		93		
BMI mean change from baseline (SD), kg/m^2^	0.2	2.1	0.5	1.9	0.4	1.8	0.7	2.0	
Unadjusted Coef.	1 (reference)		0.267	0.240	0.117	0.241	0.475	0.300	0.804
Multivariate model 3 Coef. (SE) ^‡^	1 (reference)		0.159	0.237	−0.038	0.239	0.172	0.300	0.422
Multivariate model 4 Coef. (SE) ^§^	1 (reference)		0.133	0.240	−0.053	0.243	0.203	0.307	0.523

SD: Standard Deviation; SE: Standard Error; * Model 1: Multivariable-adjusted model adjusted for age, social class (manual and non-manual workers), alcohol intake (non-drinker, drinker has been divided into 3 equal groups), smokers (non-smoker, current smoker, previous smoker), leisure activity (yes and no), total energy intake. ^†^ Model 2: Model 1 and additionally adjusted for sugar intake, red meat intake and egg intake. ^‡^ Model 3: Model 1 and additionally adjusted for BMI at baseline. ^§^ Model 4: Model 2 and additionally adjusted for BMI at baseline, incident of diabetes, cancer, hypertension and cardiovascular disease during 10-year following up.

**Table 4:** Cross-sectional analysis of BMI at baseline and longitudinal analyses over 10-year of BMI changes from baseline by quartile of cheese intake.

	Cheese Intake (g/day)								*p* for Trend
	0 ≤ Cheese < 11		11 ≤ Cheese < 16		16 ≤ Cheese < 22		22 ≤ Cheese < 130		
Cross-sectional analyses									
Participants, *n*	368		426		459		411		
BMI mean (SD), kg/m^2^	26.1	3.9	26.1	3.4	26.1	3.3	26.6	3.4	
Unadjusted Coef. (SE)	1 (reference)		0.032	0.247	0.051	0.243	0.510	0.249	0.046
Multivariate model 1 Coef. (SE) *	1 (reference)		0.085	0.244	0.156	0.243	0.627	0.254	0.015
Multivariate model 2 Coef. (SE) ^†^	1 (reference)		0.021	0.244	0.078	0.244	0.515	0.255	0.045
Longitudinal analysis at 5-years									
Participants, *n*	364		424		456		408		
BMI mean change from baseline (SD), kg/m^2^	0.4	1.4	0.2	1.5	0.3	1.4	0.1	1.5	
Unadjusted Coef. (SE)	1 (reference)		−0.134	0.105	−0.099	0.104	−0.290	0.106	0.014
Multivariate model 3 Coef. (SE) ^‡^	1 (reference)		−0.139	0.105	−0.114	0.105	−0.297	0.110	0.014
Multivariate model 4 Coef. (SE) ^§^	1 (reference)		−0.126	0.106	−0.119	0.106	−0.293	0.110	0.013
Longitudinal analysis at 10-years									
Participants, *n*	311		361		375		351		
BMI mean change from baseline (SD), kg/m^2^	0.5	1.9	0.6	1.9	0.4	1.8	0.3	1.8	
Unadjusted Coef. (SE)	1 (reference)		0.043	0.146	−0.164	0.144	−0.266	0.146	0.025
Multivariate model 3 Coef. (SE) ^‡^	1 (reference)		0.008	0.144	−0.113	0.145	−0.188	0.150	0.143
Multivariate model 4 Coef. (SE) ^§^	1 (reference)		0.045	0.144	−0.097	0.145	−0.202	0.150	0.109

SD: Standard Deviation; SE: Standard Error; * Model 1: Multivariable-adjusted model adjusted for age, social class (manual and non-manual workers), alcohol intake (non-drinker, drinker has been divided into 3 equal groups), smokers (non-smoker, current smoker, previous smoker), leisure activity (yes and no), total energy intake. ^†^ Model 2: Model 1 and additionally adjusted for sugar intake, red meat intake and egg intake. ^‡^ Model 3: Model 1 and additionally adjusted for BMI at baseline. ^§^ Model 4: Model 2 and additionally adjusted for BMI at baseline, incident of diabetes, cancer, hypertension and cardiovascular disease during 10-year following up.

**Table 5:** Cross-sectional analysis of BMI at baseline and longitudinal analyses over 10-year of BMI changes from baseline by tertile of cream intake.

	Cream Intake (g/day)						*p* for Trend
	0		0 < Cream < 5.4		5.4 ≤ Cream < 40.5		
Cross-sectional analysis							
Participants, *n*	1094		220		244		
BMI mean (SD), kg/m^2^	26.4	3.6	25.9	3.2	26.1	2.9	
Unadjusted Coef. (SE)	1 (reference)		−0.501	0.255	−0.243	0.244	0.137
Multivariate model 1 Coef. (SE) *	1 (reference)		−0.547	0.255	−0.091	0.245	0.339
Multivariate model 2 Coef. (SE) ^†^	1 (reference)		−0.498	0.253	−0.139	0.244	0.277
Longitudinal analysis at 5-years							
Participants, *n*	1085		220		242		
BMI mean change from baseline (SD), kg/m^2^	0.2	1.5	0.2	1.3	0.3	1.4	
Unadjusted Coef. (SE)	1 (reference)		−0.031	0.109	0.088	0.105	0.504
Multivariate model 3 Coef. (SE) ^‡^	1 (reference)		−0.051	0.110	0.051	0.106	0.761
Multivariate model 4 Coef. (SE) ^§^	1 (reference)		−0.031	0.111	0.067	0.107	0.628
Longitudinal analysis at 10-years							
Participants, *n*	904		190		211		
BMI mean change from baseline (SD), kg/m^2^	0.4	1.9	0.4	1.6	0.6	1.8	
Unadjusted Coef. (SE)	1 (reference)		−0.044	0.149	0.193	0.143	0.256
Multivariate model 3 Coef. (SE) ^‡^	1 (reference)		−0.089	0.148	0.181	0.142	0.329
Multivariate model 4 Coef. (SE) ^§^	1 (reference)		−0.096	0.148	0.176	0.142	0.348

SD: Standard Deviation; SE: Standard Error; * Model 1: Multivariable-adjusted model adjusted for age, social class (manual and non-manual workers), alcohol intake (non-drinker, drinker has been divided into 3 equal groups), smokers (non-smoker, current smoker, previous smoker), leisure activity (yes and no), total energy intake. ^†^ Model 2: Model 1 and additionally adjusted for sugar intake, red meat intake and egg intake. ^‡^ Model 3: Model 1 and additionally adjusted for BMI at baseline. ^§^ Model 4: Model 2 and additionally adjusted for BMI at baseline, incident of diabetes, cancer, hypertension and cardiovascular disease during 10-year following up.

**Table 6:** Cross-sectional analysis of BMI at baseline and longitudinal analyses over 10-year of BMI changes from baseline by quartile of butter intake.

	Butter Intake (g/day)								*p* for Trend
	0 < Butter < 12		12 < Butter < 24.3		24.3 ≤ Butter < 32.4		32.4 ≤ Butter ≤ 130		
Cross-sectional analysis									
Participants, *n*	419		431		416		357		
BMI mean (SD), kg/m^2^	26.4	3.3	25.8	3.4	26.4	3.4	26.2	3.7	
Unadjusted Coef. (SE)	1 (reference)		−0.527	0.237	0.040	0.239	−0.180	0.248	0.955
Multivariate model 1 Coef. (SE) *	1 (reference)		−0.560	0.233	0.144	0.236	0.147	0.260	0.219
Multivariate model 2 Coef. (SE) ^†^	1 (reference)		−0.434	0.233	0.229	0.236	0.184	0.260	0.167
Longitudinal analysis at 5-years									
Participants, *n*	418		426		414		353		
BMI mean change from baseline (SD), kg/m^2^	0.3	1.5	0.2	1.4	0.1	1.4	0.4	1.5	
Unadjusted Coef. (SE)	1 (reference)		−0.091	0.101	−0.198	0.102	0.097	0.106	0.709
Multivariate model 3 Coef. (SE) ^‡^	1 (reference)		−0.095	0.101	−0.192	0.102	0.071	0.113	0.998
Multivariate model 4 Coef. (SE) ^§^	1 (reference)		−0.089	0.102	−0.183	0.103	0.050	0.113	0.891
Longitudinal analysis at 10-years									
Participants, *n*	358		353		344		305		
BMI mean change from baseline (SD), kg/m^2^	0.5	1.8	0.4	1.9	0.3	1.9	0.6	2.0	
Unadjusted Coef. (SE)	1 (reference)		−0.076	0.141	−0.234	0.142	0.092	0.146	0.894
Multivariate model 3 Coef. (SE) ^‡^	1 (reference)		−0.081	0.139	−0.196	0.140	0.054	0.153	0.915
Multivariate model 4 Coef. (SE) ^§^	1 (reference)		−0.055	0.140	−0.146	0.141	0.054	0.154	0.989

SD: Standard Deviation; SE: Standard Error; * Model 1: Multivariable-adjusted model adjusted for age, social class (manual and non-manual workers), alcohol intake (non-drinker, drinker has been divided into 3 equal groups), smokers (non-smoker, current smoker, previous smoker), leisure activity (yes and no), total energy intake. ^†^ Model 2: Model 1 and additionally adjusted for sugar intake, red meat intake and egg intake. ^‡^ Model 3: Model 1 and additionally adjusted for BMI at baseline. ^§^ Model 4: Model 2 and additionally adjusted for BMI at baseline, incident of diabetes, cancer, hypertension and cardiovascular disease during 10-year following up.

## Supplementary Materials

The following are available online at http://www.mdpi.com/2072-6643/10/10/1515/s1, Table S1: Dairy consumption over 10-year following up in the Caerphilly Prospective Cohort Study.
