# Supplementary material for: Dairy Foods and Body Mass Index over 10-Year: Evidence from the Caerphilly Prospective Cohort Study
Source: Nutrients. 2018 Oct 16;10(10):1515. doi: 10.3390/nu10101515 (PMC6213600; doi:10.3390/nu10101515)
Supplement: Supplementary file 1 [file nutrients-10-01515-s001.pdf]

Supplemental Table S1. Dairy consumption over 10-year following up in the Caerphilly Prospective Cohort Study.

Dairy consumption (g/d)	Baseline		5-years		10-years		*P*-value (baseline vs. 5-year)*	*P*-value (baseline vs. 10-year) †
	Mean	SE	Mean	SE	Mean	SE		
Milk	288.4	4.5	241.7	4.6	188.7	4.7	<0.0001	<0.0001
Cheese	18.4	0.3	18.3	0.3	15.2	0.3	0.7335	<0.0001
Cream	1.7	0.1	1.4	0.1	1.3	0.1	0.004	<0.0001
Butter	24.5	0.5	17.9	0.4	11.5	0.4	0.004	<0.0001
Total dairy	333.0	4.5	279.4	4.7	216.6	4.9	<0.0001	<0.0001

SE: Standard Error;

*Data show *t*-test significance between baseline and 5-year following up.

†Data show *t*-test significance between baseline and 10-year following up.
